# A Reservoir of Drug-Resistant Pathogenic Bacteria in Asymptomatic Hosts

**DOI:** 10.1371/journal.pone.0003749

**Published:** 2008-11-18

**Authors:** Gabriel G. Perron, Sylvain Quessy, Graham Bell

**Affiliations:** 1 Department of Biology, McGill University, Montreal, Quebec, Canada; 2 Département de Pathologie et Microbiologie, Faculté de médecine vétérinaire, Université de Montréal, Saint-Hyacinthe, Quebec, Canada; University of Sydney, Australia

## Abstract

The population genetics of pathogenic bacteria has been intensively studied in order to understand the spread of disease and the evolution of virulence and drug resistance. However, much less attention has been paid to bacterial carriage populations, which inhabit hosts without producing disease. Since new virulent strains that cause disease can be recruited from the carriage population of bacteria, our understanding of infectious disease is seriously incomplete without knowledge on the population structure of pathogenic bacteria living in an asymptomatic host. We report the first extensive survey of the abundance and diversity of a human pathogen in asymptomatic animal hosts. We have found that asymptomatic swine from livestock productions frequently carry populations of *Salmonella enterica* with a broad range of drug-resistant strains and genetic diversity greatly exceeding that previously described. This study shows how agricultural practice and human intervention may lead and influence the evolution of a hidden reservoir of pathogens, with important implications for human health.

## Introduction

Virulent strains of bacteria damage their hosts by disrupting vital functions while using host resources to fuel their own reproduction [1]. A high level of virulence is likely to evolve when the transmission time (between the initial infection and the release of new infective cells from the host) is short, since rapidly reproducing strains of the pathogen outcompete the slow grower, thus utilising more of the host resources. Longer transmission times tend to favour strains that achieve greater overall reproduction through the prudent use of host resources, leading to lower virulence and greater host survival [2], [3]. Virulent strains of human and animal pathogens are controlled by vaccines or drugs such as antibiotics, while bacterial populations, in contrast, readily evolve resistance [4]. Although antibiotic resistance can sometimes be associated with plasmids conferring growth rate advantages in some environments [5], in the absence of antibiotics, resistant strains are usually counterselected because they grow more slowly as a result of the antagonistic pleiotropic cost of resistance on fitness [6]. Hence, the evolution of virulent strains resistant to drugs is governed by two trade-offs: between the rate of reproduction and host morbidity, and between growth rate and drug resistance.

The outcome of selection, under these two constraints, will depend on the availability of genetic variation [7]. The overall diversity and the population structure of pathogenic bacteria are central to understanding the evolution of infectious disease, and have been extensively studied [8]–[10]. Precisely because virulence and antibiotic resistance are important to public health, these studies have largely concerned strains isolated from individuals showing symptoms of disease. Many pathogenic bacteria, however, do not require the induction of overt disease to reproduce and disperse effectively [11]. It was for this reason that Maynard Smith et al [9] advocated the study of carriage populations to understand the origin of new virulent strains.

Despite the growing concerns about carrier populations of pathogenic and drug-resistant bacteria, little information is available for any of these pathogens. Where the genetic diversity of human pathogens has been intensively studied, for example in *Staphylococcus aureus* [12] and *Neisseria meningitides* [13] (two human commensal bacteria which cause sporadic outbreaks of disease), the number of studies focusing on disease isolates far outnumbers those of the more diverse carrier populations. The same is true in the veterinary and food safety literature. Since the more virulent strains of pathogens are associated with the growing threat from foodborne infections, [14] their study has dominated most of our efforts to understand bacterial population structure in animal hosts. However, it is now understood that many infections will persist with no apparent sign of disease in the animal while producing symptoms in humans. In *Escherichia coli* O157:H7 for example, the Stx toxin is not virulent in cattle, but may harm a human host [14]. Therefore, human consumption of meat contaminated by this toxin represents a risk of food-borne infection [14], [15]. To properly design control strategies and predict the spread of infectious diseases, it is essential to appreciate the full diversity of a bacteria population, since this latter drives the evolution and maintenance of virulence and drug resistance.

*Salmonella* enterica is one of the most important food-borne infections in developed countries [16] and therefore is one of the primary interests of food safety control and surveillance programs in many countries [17]–[21]. However, despite its high prevalence in disease, little is known about the biology of the bacterium in asymptomatic hosts. As the importance of asymptomatic strains of *Salmonella* has only recently been recognised [18], [20], [22], [23], the bacterium offers exceptional opportunities to investigate the evolution of pathogenic bacteria in the context of a broader ecology. Using a collection of *Salmonella* strains isolated from several thousand carcasses of asymptomatic animals (excluding all isolates from farms with a history of clinical *Salmonella* infection), we identified the patterns and the changes in population structure and the genetic diversity of *Salmonella* and comment on the possible impact of public health intervention on the evolution of pathogenic bacteria. Using a much smaller collection of strains, we previously demonstrated that *Salmonella* strains isolated from an asymptomatic host have greater genetic diversity than clinical isolates collected from humans [22]. However, the previous study focused on a specific variant of the *Salmonella* genus (i.e. Salmonella Typhimurium DT104) and did not document the full diversity associated with *Salmonella* in asymptomatic hosts nor investigated the evolutionary causes for such difference in diversity. Here, we report the first extensive survey of the abundance and genetic diversity of a human pathogen in an asymptomatic animal host and clearly demonstrate the importance of studying carrier populations. By using comparative studies, we are able to show the potential impact of public health interventions on the evolution of genetic diversity and multidrug-resistance in pathogenic bacteria.

## Results

**Figure 1:**
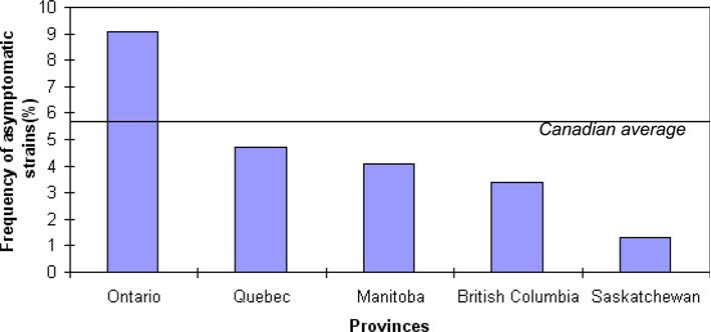
Frequency of *Salmonella* strains isolated from asymptomatic swine by territories.

We isolated *S. enterica* from about 6% of all sampled asymptomatic pigs (Figure 1). The prevalence of *Salmonella* in asymptomatic pigs varied greatly among territories (*Χ^2^* = 84.88; *d.f.* = 4; *P <* 0.0001), reaching a maximum of 9.1% in Ontario. Despite the difference in sampling effort among territories, the abundance of infected but asymptomatic hosts in all provinces represents a serious threat to food safety because positive animals showing no clinical signs of infection can enter the food chain [24]. Furthermore, asymptomatic carriers can have a significant role in the contamination of the environment and other animals, since large volumes of the bacterium can be excreted during fattening, transport and slaughter [25]. The most frequently isolated serotype (44% of all *Salmonella* isolates; Table S1) was Typhimurium and the most frequent lysotype (50% of Typhimurium isolates; Table S2) was DT104. This is consistent with the composition of pathogenic populations: Typhimurium DT104 has been the most important subtype identified in animal and human disease since it was first recorded in the mid-1990s [20], [26]. This consistency between the sampled asymptomatic population of bacteria and the disease-associated population suggests that asymptomatic animals could be an important reservoir for human pathogens.

**Figure 2:**
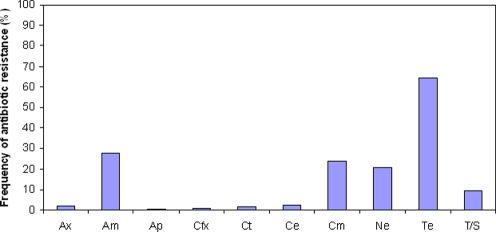
Frequency of antibiotic-resistant strains of *Salmonella* among isolates from asymptomatic swine. Antibiotics are: Ax, co-amoxiclav; Am, ampicillin; Ap, apramycin; Cfx, cefoxitin; Ct, ceftiotur; Ce, cefalotin; Cm, chloramphenicol; Ne, neomycin; Te, tetracycline; TS, trimethoprim-sulfas.

We tested 10 antibiotics and found that none were effective against all our isolates. The incidence of resistant strains varied widely between about 1% (apramycin, cefoxitin) and 65% (tetracycline) (Figure 2). Resistance to antimicrobials has been associated with increased hospitalization and mortality rates in people and animals due to treatment failures and persistence of infection [15]. For example, treatment of severe Typhimurium infection transmitted from asymptomatic swine to people with the current broad-spectrum antibiotics, such as ampicillin (a drug still used to treat salmonellosis [27]), would result in treatment failure in more than 50% of all cases. Moreover, most strains were resistant to several antibiotics. Strikingly, 90% of Typhimurium DT104 isolates were resistant to two or more of the antibiotics tested, while up to 25% of all isolates were multidrug-resistant. In all, a total of 95 different resistant phenotypes from 391 *Salmonella* isolates were recorded in this study (Table S3). Such an extent and diversity of antimicrobial resistance in *Salmonella* from asymptomatic hosts may be a major factor in the spread of resistant strains to humans and livestock (see [15], [28]).

**Figure 3:**
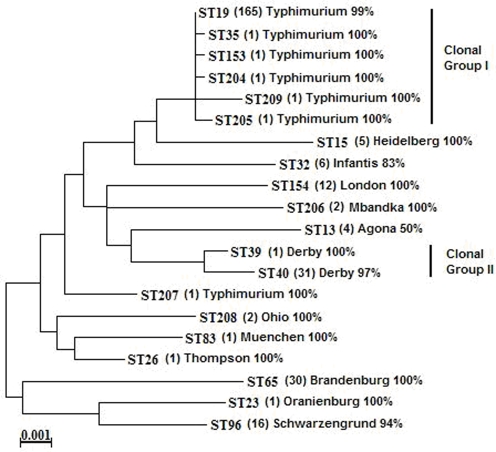
Maximum likelihood tree representing the twenty sequence types associated with asymptomatic swine. The number of isolates (in brackets) and the percentage of the most frequent serotype are shown.

We then examined the population structure and underlying genetic variation in our sample using the nucleotide sequences of seven housekeeping loci analysed by multilocus sequence typing (MLST). We identified 20 genotypes, with most falling into two clonal groups as defined by an eBURST analysis (Figure 3). We found that there was very little genetic recombination. The overall mean recombination rate was 5.4×10^−4^ per locus, with any given nucleotide being about 15 times more likely to be changed by mutation as by recombination (see Table S4). Hence, *Salmonella* from asymptomatic infections has a nearly completely clonal structure, as has been previously established for virulent strains [29]. However, while previous investigations of *Salmonella* population structure found little genetic variability over years [30], [31]or among disease-associated stains of different host origin [32], we discovered six new genotypes of DT104 among asymptomatic isolates. Furthermore, this diversity was sampled in a single host species over a period of a few weeks, and hence does not include temporal variation. Because of the clonal nature of *Salmonella*, most of the variation observed, and therefore the adaptation of the bacterium, would be transmitted vertically from novel mutations. Interestingly, the majority of the antibiotic-resistant and multidrug-resistant phenotypes were associated with this emergent diverse group of Salmonella DT104 strains. Although, antibiotic resistance can be transmitted horizontally in bacteria such as *Salmonella* [33]–[36], it has been previously demonstrated that the capability to exchange such genetic material is restricted to certain lineages [37]. Thus, it appears that despite some level of horizontal transfer among closely related strains, the distribution of traits (like antibiotic resistance) is clonally distributed and mainly affected by the evolution within clonal group.

## Discussion

We here show that asymptomatic animal hosts can be an important reservoir of human pathogenic bacteria, such as *Salmonella enterica*. Although DT104 was initially associated with high virulence in both people and animals [26], our results suggest that *S. enterica* has evolved during its passage in asymptomatic animals. The genetic diversity observed in clonal group 1 is caused mainly by changes in a single diversifying lineage of DT104 isolates, and the short genetic distances imply that this distinctive diversification was recent, a pattern predicted in adaptive radiation [38]–[40]. Furthermore, eBURST analyses, which can reliably predict the founding genotype of a group in clonal organisms [41], identified genotype ST19 (Figure 2) to be the founding genotype of clonal group 1. Genotype ST19 was the only MLST genotype previously described in the literature for *S*. Typhimurium. Although it is possible than the immune system of livestock has changed to become less susceptible to the ubiquitous DT104, the extent of genetic change detected in the bacterium suggest that diversification within the bacterial community is much more important. A similar population structure could be observed if DT104 was initially avirulent in swine or could cause disease only sporadically. However, previous data suggest that DT104 was transmitted to swine from cattle as a virulent pathogen causing severe infections in pigs [26], which supports the adaptive radiation hypothesis.

The previously unnoticed genetic diversity observed in asymptomatic *Salmonella* may be attributable to the effect of farming practices on the relative reproductive success of bacterial strains. While outbreaks of *Salmonella* in swine herds will trigger immediate attention and, in general, treatment with antibiotics, only few authorities (e.g. Denmark and Quebec) have paid attention to the control of asymptomatic carriage in animal populations. Hence, attenuated strains that cause only mild symptoms of disease, or no symptoms, will be favoured because they are much less at risk from culling or antibiotic therapy. Consequently, these strains will have a long transmission period that favours reduced virulence. Within any individual co-infected host, a virulent strain may have a short-term advantage and tend to displace a less virulent competitor, but its overall fitness is reduced when the host is treated with antibiotics or removed from the population. Selection for antibiotic resistance will be less intense in these attenuated strains.

Nevertheless, in several cases high levels of resistance may persist among asymptomatic strains of bacteria. First, low concentrations of antibiotics are often administered to infected herds as a growth promoter, or even at therapeutic levels to prevent transmission of disease to other animals [42], [43]. Such weak selective pressures are enough to maintain a trait in the population or to select for resistance to higher concentrations of antibiotics [44]. Second, restoration of normal rates of growth in resistant strains is more often attributable to compensatory mutations at other loci, rather than to back-mutation, thus preserving the resistance trait in the bacterial population [6]. Other mechanisms could explain the presence of antibiotic resistant bacteria in farm environments when no antibiotics are in current use, such as the spatial structure in a metapopulation context [45]. If antibiotic resistance is only weakly deleterious, resistant stains could be maintained in a structured environment with low or intermediate rates of immigration as long as episodic selective pressures halt the decay of these strains. As many resistance genes protect bacterial cells against other compounds found in the environment [45] or can be associated with other selective traits [5], selective pressures for antibiotic resistance can operate in the absence of antibiotics.

It is important to note that although the diversity observed in the described strains seems to have originated from point mutations, it is impossible to dismiss the impact of recombination in the generation of genetic diversity. Although it has been suggested that most bacteria, including *Salmonella*, can undergo homologous recombination (that is, the exchange of DNA between related strains of bacteria [9], [46]), our results are supportive of a clonal structure which has long been associated with *Salmonella*. On the other hand, our data also give some support to the hypothesis that hypermutable genotypes are often associated with pathogenic bacteria and antimicrobial resistance [47]. As the bulk of antimicrobial resistance is mainly associated with the most genotypically diverse group in our dataset, it would be interesting in future investigations to determine if the antibiotic resistant DT104 has an elevated mutation rate. Finally, the absence of homologous recombination in the housekeeping genes used in this study does not preclude non-homologous recombination among accessory genes or through plasmids. For example it is well know that antibiotic resistance genes can spread horizontally through integrons, phage or plasmids. However, as shown in Perron *et al.* [37], multidrug-resistance is clustered in few genotypes or lineages, suggesting that only specific serotypes/genotypes have the ability to acquire resistance genes horizontally [34]. One other area of possible future research would be to look at the interplay between the mutation rate and the presence of molecular markers of horizontal transfer, such as SGI1 [48] genes in *Salmonella,* in order to test their relative importance to the evolution of the bacterium.

### Conclusion

Strains that do not cause disease in swine may continue to express high levels of virulence in other hosts, such as humans [49]. As far as we know, this is the first extensive survey of the diversity and abundance associated with asymptomatic populations of pathogenic bacteria which may fuel the evolution of human pathogens. Our results justify the speculation of Maynard Smith et al [9] that the carriage population is an important factor in disease evolution. The genetic diversity of this carriage population permits it to evade host surveillance systems, retain or evolve resistance to antimicrobial agents, and adapt rapidly to new hosts (see Levin *et al.* [8]). Our study underlines the importance of ecological and evolutionary factors in public health issues such as the spread of food-borne pathogenic bacteria and antimicrobial resistance.

## Materials and Methods

### Bacterial strains

For a complete description of the methodology concerning the isolation and the characterisation of the strains see [22], [37]. Briefly, all *Salmonella* isolates were collected from the mesenteric lymph nodes from the carcasses of pigs showing no sign of infections. Overall, we sampled 7,441 animals over five Canadian provinces. Isolates from animals or farms with a history of infections were discarded. Serotyping and subtyping of the strains were performed at the Laboratoire d'épidémiosurveillance animale du Québec in Saint-Hyacinthe and at Health Canada Laboratory for Foodborne Zoonoses, Guelph, Ontario. In this study, S. Typhimurium var. Copenhagen isolates were combined to S. Typhimurium. To compare the prevalence of *Salmonella* strains among provinces we used the Pearson's *Χ^2^* in a 5×2 contingency table analysis where rows = provinces and columns = presence v. absence. We used the multilocus sequence typing (MLST) schemed described in Kidgell *et al.* [50] to genetically type a subsample of 282 bacterial *Salmonella* strains. The sequencing was performed by Genome Québec Innovation Centre, Montreal, Quebec.

Susceptibility to ten antibiotics was tested for all bacteria strains: co-amoxiclav, ampicillin, apramycin, cefoxitin, ceftiotur, cefalotin, chloramphenicol, enrofloxacin, gentamicin, neomycin, tetracycline and trimethoprim-sulfas. Experimentation and the interpretation of the results were conducted according to the criteria established by the CLSI (National Committee for Clinical Laboratory Standards NCCLS, 2002). Isolates with an intermediate phenotype were interpreted as susceptible in order to not over-estimate the occurrence of resistance.

### Population genetics

Population structure was tested using eBURST (http://eburst.mlst.net) as implemented by Feil et al. [51]. We constructed a Maximum Likelihood tree based on the TBR with a gamma correction of 0.015 using PAUP*. The model was chosen on the basis of the Akaike information Criterion. To test whether the frequency spectrum of mutations conformed to the expectations of the standard neutral model, we calculated the values of three test statistics: Tajima's D statistic [52], Fu's Fs [53]; and dN/dS which measures the ratio of the non-synonymous to the synonymous substitution rate. Recombination within housekeeping genes was tested using linkage disequilibrium estimated between loci [29], a coalescent-based likelihood permutation test, LDhat [54] and the PHI test [55]. All calculations were performed with either DnaSP v4.10.7 [56], LDhat v2.0 [54], SplitsTree4 [57] or through the START2 package [58] and are presented in the supplementary information.

## Supporting Information

Table S1 Distribution of *Salmonella enterica* serotypes in Canada
(0.06 MB DOC)

Table S2 Distribution of *Salmonella* Typhimurium lysotypes in Canada
(0.06 MB DOC)

Table S3 Antimicrobial resistance by serotypes of asymptomatic *Salmonella enterica* in Canada
(0.10 MB DOC)

Table S4 Population genetics analyses of MLST data of asymptomatic *Salmonella enterica*.
(0.04 MB DOC)
