# Supplementary material for: A Reservoir of Drug-Resistant Pathogenic Bacteria in Asymptomatic Hosts
Source: PLoS One. 2008 Nov 18;3(11):e3749. doi: 10.1371/journal.pone.0003749 (PMC2581806; doi:10.1371/journal.pone.0003749)
Supplement: Table S1 — Distribution of Salmonella enterica serotypes in Canada (0.06 MB DOC) [file pone.0003749.s001.doc]

| Table S1 Distribution of *Salmonella* *enterica* serotypes in Canada | | | | | | | | | | | | |
| --- | --- | --- | --- | --- | --- | --- | --- | --- | --- | --- | --- | --- |
| Serotypes | Quebec ^a^ | | Ontario ^a^ | | Manitoba | | Sask. ^a^ | | B.-C. ^a^ | | Total ^a^ | |
| Typhimurium | 78 | (53.4) | 82 | (55.4) | 9 | (11.7) | 3 | (42.3) | 0 | (0.0) | 172 | (44.3) |
| Derby | 37 | (25.3) | 19 | (9.5) | 7 | (9.1) | 2 | (28.6) | 10 | (100) | 75 | (18.6) |
| Brandenburg | 23 | (17.8) | 24 | (16.2) | 8 | (10.4) | 0 | (0.0) | 0 | (0.0) | 55 | (14.1) |
| Schwarzengrund | 2 | (1.4) | 2 | (1.4) | 32 | (41.6) | 0 | (0.0) | 0 | (0.0) | 36 | (9.3) |
| London | 0 | (0.0) | 12 | (8.1) | 4 | (5.2) | 0 | (0.0) | 0 | (0.0) | 16 | (4.1) |
| Heidelberg | 2 | (1.4) | 3 | (2.0) | 5 | (6.5) | 2 | (28.6) | 0 | (0.0) | 12 | (3.1) |
| Infantis | 0 | (0.0) | 0 | (0.0) | 6 | (7.8) | 0 | (0.0) | 0 | (0.0) | 6 | (1.5) |
| Agona | 1 | (<1) | 0 | (0.0) | 3 | (3.4) | 0 | (0.0) | 0 | (0.0) | 4 | (1.0) |
| Livingstone v. 14+ | 0 | (0.0) | 3 | (2.2) | 0 | (0.0) | 0 | (0.0) | 0 | (0.0) | 3 | (<1) |
| Ohio | 3 | (2.1) | 0 | (0.0) | 0 | (0.0) | 0 | (0.0) | 0 | (0.0) | 3 | (<1) |
| Mbandaka | 0 | (0.0) | 0 | (0.0) | 2 | (2.6) | 0 | (0.0) | 0 | (0.0) | 2 | (<1) |
| Bradford | 0 | (0.0) | 0 | (0.0) | 1 | (1.3) | 0 | (0.0) | 0 | (0.0) | 1 | (<1) |
| Montevideo | 0 | (0.0) | 1 | (<1) | 0 | (0.0) | 0 | (0.0) | 0 | (0.0) | 1 | (<1) |
| Muenchen | 0 | (0.0) | 1 | (<1) | 0 | (0.0) | 0 | (0.0) | 0 | (0.0) | 1 | (<1) |
| Newport | 0 | (0.0) | 1 | (<1) | 0 | (0.0) | 0 | (0.0) | 0 | (0.0) | 1 | (<1) |
| Oranienburg | 0 | (0.0) | 1 | (<1) | 0 | (0.0) | 0 | (0.0) | 0 | (0.0) | 1 | (<1) |
| Stanley | 0 | (0.0) | 1 | (<1) | 0 | (0.0) | 0 | (0.0) | 0 | (0.0) | 1 | (<1) |
| Thompson | 1 | (<1) | 0 | (0.0) | 0 | (0.0) | 0 | (0.0) | 0 | (0.0) | 1 | (<1) |
| Total b | 147 | (37.6) | 150 | (38.1) | 77 | (19.8) | 7 | (1.8) | 10 | (2.6) | 391 | |
| ^a^ Data is presented as prevalence and percent within location. | | | | | | | | | | | | |
| ^b^ Data is presented as total prevalence and percent from total *Salmonella* observation. | | | | | | | | | | | | |
| Sask., Saskatchewan; B.-C., British-Columbia. | | | | | | | | | | | | |
