# Supplementary material for: A Reservoir of Drug-Resistant Pathogenic Bacteria in Asymptomatic Hosts
Source: PLoS One. 2008 Nov 18;3(11):e3749. doi: 10.1371/journal.pone.0003749 (PMC2581806; doi:10.1371/journal.pone.0003749)
Supplement: Table S2 — Distribution of Salmonella Typhimurium lysotypes in Canada (0.06 MB DOC) [file pone.0003749.s002.doc]

| Table S2 Distribution of *Salmonella* Typhimurium lysotypes in Canada | | | | | | | | | | | | |
| --- | --- | --- | --- | --- | --- | --- | --- | --- | --- | --- | --- | --- |
| Lysotype | Quebec ^a^ | | Ontario ^a^ | | Manitoba | | Sask. ^a^ | | B.-C. ^a^ | | Total ^a^ | |
| Lys 104 | 22 | (28.2) | 36 | (43.9) | 2 | (22.2) | 0 | (0.0) | 0 | (0.0) | 60 | (34.9) |
| Lys 208 | 2 | (2.6) | 14 | (17.1) | 0 | (0.0) | 0 | (0.0) | 0 | (0.0) | 16 | (9.3) |
| Lys 193 | 14 | (17.9) | 1 | (1.2) | 0 | (0.0) | 0 | (0.0) | 0 | (0.0) | 15 | (8.7) |
| Lys 12 | 9 | (11.5) | 5 | (6.1) | 0 | (0.0) | 0 | (0.0) | 0 | (0.0) | 14 | (8.1) |
| Lys 104a | 9 | (11.5) | 5 | (6.1) | 0 | (0.0) | 0 | (0.0) | 0 | (0.0) | 14 | (8.1) |
| Lys 104b | 0 | (0.0) | 11 | (13.4) | 0 | (0.0) | 0 | (0.0) | 0 | (0.0) | 11 | (6.4) |
| Lys 170 | 3 | (3.8) | 4 | (4.9) | 3 | (33.3) | 0 | (0.0) | 0 | (0.0) | 10 | 5.8) |
| Lys 108 | 3 | (3.8) | 2 | (2.4) | 3 | (33.3) | 0 | (0.0) | 0 | (0.0) | 8 | (4.7) |
| U302 | 7 | (9.0) | 1 | (1.2) | 0 | (0.0) | 0 | (0.0) | 0 | (0.0) | 8 | (4.7) |
| Lys 208 var. | 5 | (6.4( | 0 | (0.0) | 0 | (0.0) | 0 | (0.0) | 0 | (0.0) | 5 | (2.9) |
| Lys 110B | 2 | (2.6) | 0 | (0.0) | 1 | (11.1) | 0 | (0.0) | 0 | (0.0) | 3 | (1.7) |
| Lys 22 | 0 | (0.0) | 0 | (0.0) | 0 | (0.0) | 3 | (100) | 0 | (0.0) | 3 | (1.7) |
| Lys 120 | 1 | (1.3) | 1 | (1.2) | 0 | (0.0) | 0 | (0.0) | 0 | (0.0) | 2 | (1.2) |
| Lys 35 | 0 | (0.0) | 1 | (1.2) | 0 | (0.0) | 0 | (0.0) | 0 | (0.0) | 1 | (1.2) |
| UT8 | 0 | (0.0) | 1 | (1.2) | 0 | (0.0) | 0 | (0.0) | 0 | (0.0) | 1 | (<1) |
| Untypable | 1 | (1.3) | 0 | (0.0) | 0 | (0.0) | 0 | (0.0) | 0 | (0.0) | 1 | (<1) |
| Total ^b^ | 78 | (45.3) | 82 | (47.7) | 9 | (5.2) | 3 | (1.7) | 0 | (0.0) | 172 | |
| ^a^ Data is presented as prevalence and percent within location. | | | | | | | | | | | | |
| ^b^ Data is presented as total prevalence and percent from total Typhimurium. | | | | | | | | | | | | |
| Sask., Saskatchewan; B.-C., British-Columbia. | | | | | | | | | | | | |
