# Supplementary material for: A Reservoir of Drug-Resistant Pathogenic Bacteria in Asymptomatic Hosts
Source: PLoS One. 2008 Nov 18;3(11):e3749. doi: 10.1371/journal.pone.0003749 (PMC2581806; doi:10.1371/journal.pone.0003749)
Supplement: Table S3 — Antimicrobial resistance by serotypes of asymptomatic Salmonella enterica in Canada (0.10 MB DOC) [file pone.0003749.s003.doc]

| Table S3 Antimicrobial resistance by serotypes of asymptomatic *Salmonella enterica* in Canada | | | | | | | | | | | | | | | | | | | | | |
| --- | --- | --- | --- | --- | --- | --- | --- | --- | --- | --- | --- | --- | --- | --- | --- | --- | --- | --- | --- | --- | --- |
| Lysotype /  Location | n | Ax | | Am | | Ap | | Cfx | | Ct | | Ce | | Cm | | Ne | | Te | | T/S | |
| Typhimurium |  |  |  |  |  |  |  |  |  |  |  |  |  |  |  |  |  |  |  |  |  |
| Quebec ^a^ | 78 | 0 | (0.0) | 44 | (56.4) | 0 | (0.0) | 0 | (0.0) | 0 | (0.0) | 0 | (0.0) | 37 | (47.4) | 54 | (75) | 75 | (96.2) | 30 | (38.5) |
| Ontario ^a^ | 82 | 3 | (3.7) | 51 | (62.2) | 0 | (0.0) | 1 | (1.2) | 1 | (1.2) | 1 | (1.2) | 50 | (61.0) | 18 | (22.0) | 72 | (87.8) | 2 | (2.4) |
| Manitoba ^a^ | 9 | 0 | (0.0) | 1 | (11.1) | 0 | (0.0) | 0 | (0.0) | 0 | (0.0) | 0 | (0.0) | 1 | (11.1) | 0 | (0.0) | 1 | (11.1) | 0 | (0.0) |
| Sask. ^a^ | 3 | 0 | (0.0) | 0 | (0.0) | 0 | (0.0) | 0 | (0.0) | 0 | (0.0) | 0 | (0.0) | 0 | (0.0) | 0 | (0.0) | 3 | (100) | 0 | (0.0) |
| B.-C. ^a^ | 0 | - | - | - | - | - | - | - | - | - | - | - | - | - | - | - | - | - | - | - | - |
| sub-total ^a^ | 172 | 3 | (1.7) | 96 | (53.5) | 0 | (0.0) | 1 | (<1) | 1 | (<1) | 1 | (<1) | 88 | (51.2) | 72 | (41.9) | 151 | (87.8) | 32 | (18.6) |
| Derby |  |  |  |  |  |  |  |  |  |  |  |  |  |  |  |  |  |  |  |  |  |
| Quebec ^a^ | 37 | 0 | (0.0) | 0 | (0.0) | 2 | (5.4) | 0 | (0.0) | 0 | (0.0) | 0 | (0.0) | 0 | (0.0) | 5 | (13.5) | 26 | (75.0) | 2 | (5.4) |
| Ontario ^a^ | 19 | 1 | (0.0) | 3 | (15.7) | 0 | (0.0) | 1 | (5.3) | 3 | (15.7) | 3 | (15.7) | 1 | (5.3) | 0 | (0.0) | 17 | (89.5) | 1 | (5.3) |
| Manitoba ^a^ | 7 | 0 | (0.0) | 0 | (0.0) | 0 | (0.0) | 0 | (0.0) | 0 | (0.0) | 0 | (0.0) | 0 | (0.0) | 0 | (0.0) | 7 | (100) | 0 | (0.0) |
| Sask. ^a^ | 2 | 0 | (0.0) | 0 | (0.0) | 0 | (0.0) | 0 | (0.0) | 0 | (0.0) | 0 | (0.0) | 0 | (0.0) | 0 | (0.0) | 0 | (0.0) | 0 | (0.0) |
| B.-C. ^a^ | 10 | 0 | (0.0) | 1 | (10.0) | 0 | (0.0) | 0 | (0.0) | 0 | (0.0) | 0 | (0.0) | 1 | (10.0) | 1 | (10.0) | 4 | (40.0) | 0 | (0.0) |
| sub-total ^a^ | 75 | 1 | (1.3) | 4 | (5.3) | 2 | (2.7) | 1 | (10.0) | 3 | (4.0) | 3 | (4.0) | 2 | (2.7) | 6 | (8.0) | 54 | (62.7) | 3 | (4.0) |
| All other |  |  |  |  |  |  |  |  |  |  |  |  |  |  |  |  |  |  |  |  |  |
| Quebec ^a^ | 32 | 2 | (6.3) | 3 | (9.4) | 1 | (3.1) | 2 | (6.3) | 2 | (6.3) | 2 | (6.3) | 1 | (3.1) | 1 | (3.1) | 15 | (46.9) | 1 | (3.1) |
| Ontario ^a^ | 49 | 2 | (4.1) | 4 | (8.2) | 0 | (0.0) | 0 | (0.0) | 1 | (2.0) | 3 | (6.1) | 2 | (2.0) | 1 | (2.0) | 4 | (6.1) | 1 | (2.0) |
| Manitoba ^a^ | 61 | 0 | (0.0) | 1 | (16.4) | 0 | (0.0) | 0 | (0.0) | 0 | (0.0) | 0 | (0.0) | 0 | (0.0) | 0 | (0.0) | 26 | (42.6) | 0 | (0.0) |
| Sask. ^a^ | 2 | 0 | (0.0) | 0 | (0.0) | 0 | (0.0) | 0 | (0.0) | 0 | (0.0) | 0 | (0.0) | 0 | (0.0) | 0 | (0.0) | 2 | (100) | 0 | (0.0) |
| B.-C. ^a^ | 0 | - |  | - |  | - |  | - |  | - |  | - |  | - |  | - |  | - |  | - |  |
| sub-total ^a^ | 144 | 4 | (2.8) | 8 | (5.6) | 1 | (<1) | 2 | (1.4) | 3 | (2.1) | 5 | (3.5) | 3 | (1.4) | 2 | (1.4) | 47 | (31.9) | 2 | (1.4) |
| Total ^b^ | 391 | 8 | (2.0) | 108 | (27.6) | 3 | (<1) | 4 | (1.0) | 7 | (1.8) | 9 | (2.3) | 93 | (23.8) | 80 | (20.5) | 252 | (64.5) | 37 | (9.5) |
| ^a^ Data is presented as total resistant isolates and percent within location. | | | | | | | | | | | | | | | | | | | | | |
| ^b^ Data is presented as total resistant isolates and percent over total population. | | | | | | | | | | | | | | | | | | | | | |
| Sask., Sakatchewan; B.-C., British-Columbia; Ax, co-amoxiclav; Amp, ampicillin; Ap, apramycin; Cfx, cefoxitin; Ct, ceftiotur; Ce, cefalotin; Cm, chloramphenicol; Ne, neomycin; Te, tetracycline; T/S, trimethoprim-sulfas. | | | | | | | | | | | | | | | | | | | | | |
