# Supplementary material for: A Reservoir of Drug-Resistant Pathogenic Bacteria in Asymptomatic Hosts
Source: PLoS One. 2008 Nov 18;3(11):e3749. doi: 10.1371/journal.pone.0003749 (PMC2581806; doi:10.1371/journal.pone.0003749)
Supplement: Table S4 — Population genetics analyses of MLST data of asymptomatic Salmonella enterica. (0.04 MB DOC) [file pone.0003749.s004.doc]

Table S4 Population genetics analyses of MLST data of asymptomatic *Salmonella enterica*.

| Gene | Length | No. of | θ*_W_* | π | selection | | |  | recombination | |
| --- | --- | --- | --- | --- | --- | --- | --- | --- | --- | --- |
|  | (bp) | alleles |  |  | Tajima’s D | Fu’s Fs | dN/dS |  | ρ | ε |
| *aro*C | 501 | 12 | 0.0045 | 0.00451 | -0.15648 | -0.094 | 0.0274 |  | 0.000 | 0.000 |
| *dna*N | 501 | 12 | 0.0061 | 0.00482 | -0.53994 | 0.220 | 0.0208 |  | 0.000 | 0.000 |
| *hem*D | 432 | 13 | 0.0061 | 0.00759 | 0.33624 | 1.273 | 0.2260 |  | 0.0021 | 0.276 |
| *hisD* | 501 | 10 | 0.0084 | 0.01284 | 1.44838 | 10.758 | 0.0223 |  | 0.0009 | 0.070 |
| *pur*E | 399 | 10 | 0.0081 | 0.00723 | -0.26738 | 2.363 | 0.0229 |  | 0.000 | 0.000 |
| *suc*A | 501 | 16 | 0.0071 | 0.00571 | -0.50212 | -0.957 | 0.0084 |  | 0.0005 | 0.087 |
| *thr*A | 501 | 14 | 0.0074 | 0.00762 | -0.13697 | 1.631 | 0.000 |  | 0.0013 | 0.170 |
| Mean | 476.6 | 12.4 | 0.0193 | 0.00822 | -0.33044 | 2.7671 | 0.5239 |  | 0.0005 | 0.068 |
| Concat. | 3,336 | 20 | 0.0060 | 0.00612 | - | - | - |  | 0.000 | 0.0039 |
